# Stratifying Multiple Sclerosis Susceptibility Risk: The Role of HLA‐E*01 and Infectious Mononucleosis in a Population Cohort

**DOI:** 10.1111/ene.70131

**Published:** 2025-04-07

**Authors:** Andrea Nova, Davide Gentilini, Giovanni Di Caprio, Sonia Bourguiba‐Hachemi, Nicolas Vince, Pierre‐Antoine Gourraud, Luisa Bernardinelli, Teresa Fazia

**Affiliations:** ^1^ Department of Brain and Behavioral Sciences University of Pavia Pavia Italy; ^2^ Bioinformatics and Statistical Genomic Unit IRCCS Istituto Auxologico Italiano Milan Italy; ^3^ Center for Research in Transplantation and Translational Immunology, UMR 1064 Nantes Université, CHU Nantes, INSERM Nantes France

**Keywords:** EBV, HLA, MHC, mononucleosis, multiple sclerosis

## Abstract

**Background:**

Epstein–Barr Virus (EBV) and its clinical manifestation, infectious mononucleosis (IM), are strongly linked to MS risk. A recent in vitro study suggests that HLA‐E*01:03, in contrast to HLA‐E*01:01, may protect against MS through more effective immune responses against EBV‐infected B cells. However, the role of HLA‐E*01 in MS remains unclear.

**Methods:**

We investigated if HLA‐E*01:01 was significantly associated with higher MS risk in individuals with a history of IM diagnosis, using 487,144 individuals from the UK Biobank's cohort. We estimated the interaction between HLA‐E*01:01 and IM using Cox proportional hazard models, adjusting for demographics, smoking, childhood body size, older siblings, and MS‐related HLA alleles (e.g., HLA‐DRB1*15:01).

**Results:**

HLA‐E*01:01 allele alone was not significantly associated with IM or MS (*p* > 0.05). However, a significant interaction between HLA‐E*01:01 and IM history was observed in relation to MS risk (*p* < 0.001). Specifically, MS risk was significantly higher in both HLA‐E*01:01 heterozygotes (HR = 1.74 [95% CI: 1.36, 1.97], *p* < 0.001) and homozygotes (HR = 3.01 [95% CI: 1.81, 3.88], *p* < 0.001) with IM history, compared to HLA‐E*01:03 homozygotes*.* Conversely, these associations were non‐significant in individuals without IM history (*p* > 0.05). The estimated proportion of the combined risk attributable to interaction effects was 40% in HLA‐E*01:01 heterozygotes and 65% in HLA‐E*01:01 homozygotes.

**Conclusions:**

HLA‐E*01:01 carriers diagnosed with IM are at significantly increased risk of MS, independently from other MS‐related HLA alleles. This supports the hypothesis that HLA‐E*01:01 may contribute to MS susceptibility due to weaker immune control over EBV infection. Incorporating HLA‐E*01:01 into existing MHC‐based MS risk models could then enhance personalized risk assessments in individuals with IM history.

## 1 Introduction

Multiple sclerosis (MS) is a complex autoimmune disease of the central nervous system, influenced by genetic susceptibility, environmental exposures, and immune system interactions [1, 2, 3]. Epstein–Barr virus (EBV) infection is a key environmental trigger [4, 5, 6, 7], with infectious mononucleosis (IM), its symptomatic manifestation, being strongly associated with increased MS risk [8, 9]. However, given that EBV infects over 90% of the global adult population, yet only a fraction develops MS, it is likely that host genetics play a key role in determining disease susceptibility. In this context, several studies highlighted how the presence of the MS‐deleterious allele HLA‐DRB1*15:01 and the absence of the MS‐protective HLA‐A*02:01 synergistically amplify MS risk in EBV‐infected individuals with IM history [10, 11, 12].

More recently, attention has shifted to *HLA‐E* polymorphisms and their influence on immune responses to EBV [5]. *HLA‐E* is a non‐classical HLA class I molecule with various functions in both innate and adaptive immunology, including natural killer cell modulation via interaction with the CD94/NKG2 receptors [13]. Unlike classical HLA class I genes, *HLA‐E* polymorphism is low in frequency and occurs with two variants, HLA‐E*01:01 and HLA‐E*01:03. In general, studies have shown that the predominant presence of the HLA‐E*01:03 allele can provide higher molecular expression of *HLA‐E* at the cell surface than the HLA‐E*01:01 allele [14]. A recent study by Vietzen et al. [5] found the HLA‐E*01:03/01:03 genotype (homozygous) to be overrepresented in individuals with high Epstein–Barr nuclear antigen 1 (EBNA1) antibody levels who did not develop MS. Furthermore, an in vitro co‐culture experiment showed that the HLA‐E*01:03 allele, in contrast to HLA‐E*01:01, enhances immune control over EBV‐infected B cells through potent HLA‐E‐restricted CD8+ T cell responses, potentially preventing MS‐related autoimmune processes. However, given the complexity of the major histocompatibility complex (MHC) region and the emphasis on HLA genes relevant to transplantation, the role of HLA‐E*01 in MS risk remains largely unexplored. Notably, HLA‐E*01 has not been found to be associated with MS risk in genome‐wide association studies (GWASs) [15]. Based on the findings by Vietzen et al. [5], we hypothesized that the association between HLA‐E*01 and MS risk may be modified by the presence of a significant immune response triggered by EBV, such as the occurrence of IM [16]. This could be due to the differing abilities of HLA‐E*01:01 and HLA‐E*01:03 to control EBV infection effectively. To investigate this hypothesis, we used data from the UK Biobank, a large population‐based cohort, to examine whether HLA‐E*01 interacts with IM in modulating MS risk. IM diagnoses, obtained from medical records and self‐reports, were used as a marker of a clinically symptomatic EBV infection. Time‐to‐event analyses were then performed considering HLA‐E*01 and IM exposure, as well as other MS‐related HLA alleles (e.g., HLA‐DRB1*15:01 and HLA‐A*02:01) and key confounders such as smoking and childhood body size.

We aimed to evaluate (i) whether HLA‐E*01 is associated with IM risk, (ii) whether HLA‐E*01 influences MS risk, and (iii) whether HLA‐E*01 and IM interact to increase MS susceptibility independently of the interactions between IM and HLA‐DRB1*15:01 and HLA‐A*02:01 alleles. In particular, point (iii) seeks to determine whether individuals carrying HLA‐E*01:01 and diagnosed with IM represent a subgroup with higher MS susceptibility. Understanding this interaction could offer new insights into the gene–environment mechanisms underlying MS development and help identify individuals at higher risk based on HLA genotypes and IM history.

## 2 Methods

### 2.1 Institutional Review Board Approval

This is an observational study conducted using a population cohort identified within the UK Biobank (REC approval 11/NW/0382) [17]. All participants gave informed consent on Biobank registration and are free to withdraw from the study at any time, at which point their data are censored and cannot be included in further analyses.

### 2.2 Data

The UK Biobank offers a comprehensive range of health‐related phenotypes and genetic data collected from 502,354 individuals aged over 40 years, recruited between 2006 and 2010. The sample predominantly consists of English individuals of white ethnicity, who generally have better overall health and higher income compared to the general population [18].

### 2.3 Genotypes

In the UK Biobank, genome‐wide genotype data were collected on a large proportion of individuals in the cohort (≈97%). Genotypes were imputed using computationally efficient methods combined with the Haplotype Reference Consortium (HRC) and UK10K haplotype resource, and quality control on genetic variants were subsequently performed [19]. HLA‐E*01 was identified via the rs1264457 polymorphism, where the G allele (major) denoted the HLA‐E*01:01 variant, while the A allele (minor) denoted the HLA‐E*01:03 variant [20]. MS‐risk HLA alleles HLA‐DRB1*15:01, HLA‐DRB1*03:01, HLA‐DRB1*13:03, HLA‐DRB1*08:01, HLA‐DQA1*01:01, HLA‐DQB1*03:01, HLA‐DQB1*03:02, HLA‐B*07:02, and MS‐protective HLA alleles HLA‐A*02:01, HLA‐B*38:01, HLA‐B*44:02, and HLA‐B*55:01 were obtained by the UK Biobank performing imputation using HLA*IMP:02 [21]. MS risk HLA alleles were coded according to the number of alleles (0, 1, 2), while MS‐protective HLA alleles were coded based on presence/absence (0, 1) of the allele. Lastly, we retrieved the first four genetic principal components as calculated by the UK Biobank. We excluded from the analyses the 15,210 individuals missing genotype data, leaving 487,144 individuals with complete genotype data.

### 2.4 Infectious Mononucleosis Diagnosis

All diagnoses of IM were classified using the International Classification of Disease 10th revision (ICD‐10) B27 and were retrieved as the earliest diagnosis date reported among hospital admissions, primary care, death registries and self‐report sources (see Table S1 for further details). Self‐reported IM diagnoses were retrieved by the UK Biobank at the time of recruitment asking the participants the following two interview questions. First, “Has a doctor ever told you that you have had any other serious medical conditions or disabilities?” (the ‘other’ referring to cancer, which was discussed separately during the interview), and second, ‘In the touch screen you selected that you have been told by a doctor that you have other serious illnesses or disabilities, could you now tell me what they are?’. Therefore, IM diagnoses mainly related to cases with symptomatology severe enough to require medical attention. Using the diagnosis date, we were able to consider IM as a time‐varying exposure along the follow up.

### 2.5 Outcome

MS cases were defined according to the ICD‐10 diagnosis code G35. The diagnosis date was retrieved as the earliest date from at least one source among hospital admissions, primary care, death registries, and self‐report (see Table S1 for further details). To better characterize the date of MS onset and hence avoid delayed MS diagnoses [22], we anticipated the date of MS diagnosis in case of an earlier diagnosis of: (i) demyelination (ICD‐10: G36‐G37), (ii) abnormal findings on diagnostic imaging of the central nervous system (ICD‐10: R90), or (iii) the presence of other MS onset‐related symptoms occurring in the 5 years prior to the MS diagnosis date, such as optic neuritis (ICD‐10: H46) (see Data S2 for a complete list). Age at MS diagnosis was determined by subtracting the date of birth from the date of MS diagnosis.

### 2.6 Confounders

We considered the following confounders, collected at the initial assessment visit, for the association between IM diagnosis and MS: sex, year of birth, ethnicity (white, non‐white), place of birth, ever smoked on most or all days, childhood body size, having older siblings, and the first four genetic principal components. Using the age when the individual reported starting to smoke on most or all days, we were able to consider smoking as a time‐varying exposure during the follow‐up. UK Biobank data fields for each variable used in the analysis were provided in Table S2.

### 2.7 Statistical Analysis

Descriptive statistics were calculated for all the analyzed variables, stratified by HLA‐E*01 genotype. Missing data for the confounding variables were imputed using multiple imputation [23] (see Supporting Information). We planned the following statistical analyses based on a retrospective cohort study design. In the first analysis, we evaluated the association between HLA‐E*01 and IM diagnosis using a time‐to‐event approach. Individuals were followed from birth to the first date among IM diagnosis or date of censoring, i.e., date of death, date of MS diagnosis, date of loss to follow‐up, or date of follow‐up end (31 December 2022). HLA‐E*01 was coded as a continuous variable according to the number of HLA‐E*01:01 alleles (0, 1, 2). We implemented a Cox proportional hazards (PHs) model with age as time scale, including HLA‐E*01 as the covariate of interest, and adjusted for the other MS‐related HLA alleles, sex, year of birth, place of birth, ethnicity, having older siblings, and the first four genetic principal components. In the second analysis, using the same approach, we investigated the association between HLA‐E*01 and MS diagnosis. End of follow‐up was defined based on the first date among MS diagnosis or date of censoring, i.e., date of death, date of loss to follow‐up, or date of follow‐up end. In the third analysis, we further investigated the associations between HLA‐E*01, IM, and their interaction with MS diagnosis. To this aim, we considered IM diagnosis as a time‐varying exposure by creating a variable coded as “No” for all individuals from the start of follow‐up and changing to “Yes” from the time of IM diagnosis onward. In this analysis, we further adjusted for childhood body size and ever being a smoker (coded as a time‐varying confounder). In a Cox PH model, we included multiplicative interaction terms between IM and, respectively, HLA‐E*01, HLA‐DRB1*15, and HLA‐A*02. This method allows for adjustment for IM‐specific effects on MS due to known interaction with HLA‐DRB1*15:01 and HLA‐A*02:01 alleles. Using the Cox model's coefficient of the multiplicative interaction, we estimated the association between HLA‐E*01:01 allele and MS within subgroups of individuals with or without IM diagnosis, as well as the association between IM and MS within HLA‐E*01 genotype (see Supporting Information). Inspection of Schoenfeld residuals was conducted to assess the validity of the PH assumption, adjusting for any significant deviation including a stratification factor or using time‐varying coefficients.

We also estimated interactions between HLA‐E*01:01 allele and IM diagnosis on the additive scale, calculating the Relative Excess Risk due to Interaction (RERI) and the Attributable Proportion due to interaction (AP) (see Supporting Information). Additive interactions further help to assess if MS tends to occur only if both HLA‐E*01:01 allele and IM diagnosis are present but not if just one or the other is present, suggesting a potential biological interaction [24, 25]. We obtained 95% confidence intervals (CIs) for both multiplicative and additive interactions, implementing bootstrap sampling with replacement over 5000 replications and using the bias‐corrected accelerated method [26]. We provided two‐sided p‐values derived from 95% bootstrap CIs using the R package *boot.pval*, considering *p* < 0.05 as statistically significant.

### 2.8 Sensitivity Analyses

In the first sensitivity analysis, we evaluated if the strength of the interactions between HLA‐E*01 and IM diagnosis changed when excluding MS diagnoses over 50 years of age, i.e., late‐onset MS (LOMS) cases [27]. Individuals were followed up to the first date among MS diagnosis or date of censoring, i.e., date of death, date of loss to follow‐up, or date when the individuals reached 50 years of age. Similarly, in the second sensitivity analysis, we evaluated if the strengths of these interactions changed when excluding individuals born outside the UK (England, Wales, and Scotland).

Analyses were performed using RStudio 2023.06.1, and R code used for the main analysis was included in the Supporting Information.

## 3 Results

### 3.1 Descriptive Statistics

We analyzed a sample of 487,144 individuals. During the follow‐up period (median: 70 years, interquartile range (IQR): 64; 77), 3855 individuals (0.8%) had a diagnosis of IM and 2491 (0.5%) had a diagnosis of MS. The median age of IM diagnosis was 20 years (IQR: 17; 30), while the median age of MS diagnosis was 44 years (IQR: 36; 53). The discrepancy between this and the known average age at MS onset (≈33 years) [28] derives from the cohort's demographic structure, which included individuals born between 1934 and 1971. As the cohort aged, the absence of younger individuals born after 1971 limited the inclusion of newly diagnosed younger cases, while LOMS cases (> 50 years), whose incidence has been rising in recent years [22, 28, 29], disproportionately influenced the mean age at diagnosis at the end of follow‐up (2022).

Demographic characteristics of the overall sample and stratified by HLA‐E*01 genotypes are reported in Table 1. 78,165 individuals (16%) carried the HLA‐E*01:03/01:03 genotype, 233,129 (48%) carried the HLA‐E*01:01/01:03 genotype, and 175,850 (36%) carried the HLA‐E*01:01/01:01 genotype. Overall, individuals in the sample were more frequently females, of white ethnicity, born in England around 1950, had more frequently older siblings, and were less frequently smokers. These characteristics were nearly identical across HLA‐E*01 genotypes, while some imbalances can be found in other HLA alleles, i.e., a higher homozygotes' proportion of HLA‐DRB1*15:01, HLA‐A*02:01, HLA‐DQB1*03:02, and HLA‐B*07:02 in individuals carrying the HLA‐E*01:03/01:03 genotype, and a higher homozygotes' proportion of HLA‐DRB1*03:01 and HLA‐B*04:02 in individuals carrying the HLA‐E*01:01/01:01 genotype.

**TABLE 1:** Descriptive statistics for analyzed variables for the overall sample and stratified by HLA‐E*01 genotype.

	HLA‐E*01:03/01:03	HLA‐E*01:01/01:03	HLA‐E*01:01/01:01	Overall
	(*N* = 78,165)	(*N* = 233,129)	(*N* = 175,850)	(*N* = 487,144)
**Multiple sclerosis diagnosis**				
Yes	380 (0.5%)	1191 (0.5%)	920 (0.5%)	2491 (0.5%)
No	77,785 (99.5%)	231,938 (99.5%)	174,930 (99.5%)	484,653 (99.5%)
**Age at multiple sclerosis diagnosis**				
Median [IQR]	43.2 [35.5, 50.6]	44.9 [36.0, 54.0]	44.2 [35.9, 53.4]	44.2 [35.8, 53.3]
**Infectious mononucleosis diagnosis**				
Yes	609 (0.8%)	1901 (0.8%)	1345 (0.8%)	3855 (0.8%)
No	77,556 (99.2%)	231,228 (99.2%)	174,505 (99.2%)	483,289 (99.2%)
**Age at infectious mononucleosis diagnosis**				
Median [IQR]	20.3 [16.5, 28.7]	20.4 [16.5, 29.5]	20.2 [16.5, 29.8]	20.3 [16.5, 29.5]
**Sex**				
Females	42,696 (54.6%)	126,348 (54.2%)	95,083 (54.1%)	264,127 (54.2%)
Males	35,469 (45.4%)	106,781 (45.8%)	80,767 (45.9%)	223,017 (45.8%)
**Year of birth**				
Median [IQR]	1950 [1945, 1958]	1950 [1945, 1958]	1950 [1945, 1958]	1950 [1945, 1958]
**Ethnicity**				
Non‐White	3819 (4.9%)	9563 (4.1%)	6510 (3.7%)	19,892 (4.1%)
White	72,425 (92.7%)	219,693 (94.2%)	166,982 (95.0%)	459,100 (94.2%)
Missing	1921 (2.5%)	3873 (1.7%)	2358 (1.3%)	8152 (1.7%)
**Place of birth**				
Africa	1789 (2.3%)	4504 (1.9%)	3197 (1.8%)	9490 (1.9%)
America	1122 (1.4%)	3248 (1.4%)	2202 (1.3%)	6572 (1.3%)
Asia/Oceania	2752 (3.5%)	6208 (2.7%)	3915 (2.2%)	12,875 (2.6%)
England	60,855 (77.9%)	181,852 (78.0%)	136,361 (77.5%)	379,068 (77.8%)
Europe	1813 (2.3%)	4661 (2.0%)	2805 (1.6%)	9279 (1.9%)
Ireland/North Ireland	1011 (1.3%)	3512 (1.5%)	3249 (1.8%)	7772 (1.6%)
Scotland	5567 (7.1%)	18,214 (7.8%)	15,342 (8.7%)	39,123 (8.0%)
Wales	2974 (3.8%)	10,258 (4.4%)	8331 (4.7%)	21,563 (4.4%)
Missing	282 (0.4%)	672 (0.3%)	448 (0.3%)	1402 (0.3%)
**Have older siblings**				
No	21,243 (27.2%)	63,743 (27.3%)	47,357 (26.9%)	132,343 (27.2%)
Yes	55,698 (71.3%)	165,908 (71.2%)	125,909 (71.6%)	347,515 (71.3%)
Missing	1224 (1.6%)	3478 (1.5%)	2584 (1.5%)	7286 (1.5%)
**Childhood body size at age 10**				
Thinner	25,596 (32.7%)	76,107 (32.6%)	57,166 (32.5%)	158,869 (32.6%)
Average	38,975 (49.9%)	115,786 (49.7%)	87,611 (49.8%)	242,372 (49.8%)
Plumper	11,827 (15.1%)	36,342 (15.6%)	27,456 (15.6%)	75,625 (15.5%)
Missing	1767 (2.3%)	4894 (2.1%)	3617 (2.1%)	10,278 (2.1%)
**Ever smoked on most or all days**				
No	42,627 (54.5%)	126,747 (54.4%)	95,919 (54.5%)	265,293 (54.5%)
Yes	35,094 (44.9%)	105,185 (45.1%)	79,070 (45.0%)	219,349 (45.0%)
Missing	444 (0.6%)	1197 (0.5%)	861 (0.5%)	2502 (0.5%)
HLA‐DRB1*15:01				
Absent	53,112 (67.9%)	169,816 (72.8%)	137,809 (78.4%)	360,737 (74.1%)
Heterozygote	22,497 (28.8%)	58,342 (25.0%)	35,718 (20.3%)	116,557 (23.9%)
Homozygote	2556 (3.3%)	4971 (2.1%)	2323 (1.3%)	9850 (2.0%)
HLA‐A*02:01				
Absent	40,069 (51.3%)	125,559 (53.9%)	99,927 (56.8%)	265,555 (54.5%)
Heterozygote	31,517 (40.3%)	90,694 (38.9%)	64,824 (36.9%)	187,035 (38.4%)
Homozygote	6579 (8.4%)	16,876 (7.2%)	11,099 (6.3%)	34,554 (7.1%)
HLA‐DRB1*13:03				
Absent	76,771 (98.2%)	228,949 (98.2%)	172,719 (98.2%)	478,439 (98.2%)
Heterozygote	1388 (1.8%)	4161 (1.8%)	3106 (1.8%)	8655 (1.8%)
Homozygote	6 (0.0%)	19 (0.0%)	25 (0.0%)	50 (0.0%)
HLA‐DRB1*08:01				
Absent	75,278 (96.3%)	224,899 (96.5%)	170,026 (96.7%)	470,203 (96.5%)
Heterozygote	2859 (3.7%)	8150 (3.5%)	5761 (3.3%)	16,770 (3.4%)
Homozygote	28 (0.0%)	80 (0.0%)	63 (0.0%)	171 (0.0%)
HLA‐DQA1*01:01				
Absent	57,447 (73.5%)	171,528 (73.6%)	129,811 (73.8%)	358,786 (73.7%)
Heterozygote	18,991 (24.3%)	56,916 (24.4%)	42,488 (24.2%)	118,395 (24.3%)
Homozygote	1727 (2.2%)	4685 (2.0%)	3551 (2.0%)	9963 (2.0%)
HLA‐DQB1*03:01				
Absent	54,331 (69.5%)	159,088 (68.2%)	117,196 (66.6%)	330,615 (67.9%)
Heterozygote	21,593 (27.6%)	66,974 (28.7%)	52,484 (29.8%)	141,051 (29.0%)
Homozygote	2241 (2.9%)	7067 (3.0%)	6170 (3.5%)	15,478 (3.2%)
HLA‐DRB1*03:01				
Absent	67,476 (86.3%)	174,939 (75.0%)	114,725 (65.2%)	357,140 (73.3%)
Heterozygote	10,264 (13.1%)	54,874 (23.5%)	54,388 (30.9%)	119,526 (24.5%)
Homozygote	425 (0.5%)	3316 (1.4%)	6737 (3.8%)	10,478 (2.2%)
HLA‐DQB1*03:02				
Absent	58,536 (74.9%)	185,623 (79.6%)	149,816 (85.2%)	393,975 (80.9%)
Heterozygote	18,179 (23.3%)	45,003 (19.3%)	24,935 (14.2%)	88,117 (18.1%)
Homozygote	1450 (1.9%)	2503 (1.1%)	1099 (0.6%)	5052 (1.0%)
HLA‐B*38:01				
Absent	75,804 (97.0%)	228,343 (97.9%)	174,149 (99.0%)	478,296 (98.2%)
Heterozygote	2294 (2.9%)	4733 (2.0%)	1696 (1.0%)	8723 (1.8%)
Homozygote	67 (0.1%)	53 (0.0%)	5 (0.0%)	125 (0.0%)
HLA‐B*04:02				
Absent	71,915 (92.0%)	190,524 (81.7%)	127,969 (72.8%)	390,408 (80.1%)
Heterozygote	6113 (7.8%)	41,113 (17.6%)	43,833 (24.9%)	91,059 (18.7%)
Homozygote	137 (0.2%)	1492 (0.6%)	4048 (2.3%)	5677 (1.2%)
HLA‐B*55:01				
Absent	76,741 (98.2%)	225,689 (96.8%)	167,685 (95.4%)	470,115 (96.5%)
Heterozygote	1415 (1.8%)	7391 (3.2%)	8042 (4.6%)	16,848 (3.5%)
Homozygote	9 (0.0%)	49 (0.0%)	123 (0.1%)	181 (0.0%)
HLA‐B*07:02				
Absent	47,958 (61.4%)	165,341 (70.9%)	146,003 (83.0%)	359,302 (73.8%)
Heterozygote	26,202 (33.5%)	63,098 (27.1%)	28,432 (16.2%)	117,732 (24.2%)
Homozygote	4005 (5.1%)	4690 (2.0%)	1415 (0.8%)	10,110 (2.1%)
**Genetic principal component 1**				
Median [IQR]	−12.0 [−13.3, −10.6]	−12.2 [−13.3, −10.8]	−12.2 [−13.4, −10.9]	−12.2 [−13.3, −10.8]
**Genetic principal component 2**				
Median [IQR]	3.6 [2.4, 4.8]	3.7 [2.5, 4.8]	3.7 [2.5, 4.8]	3.7 [2.4, 4.8]
**Genetic principal component 3**				
Median [IQR]	−1.5 [−2.7, −0.2]	−1.5 [−2.7, 98.8]	−1.5 [−2.7, −0.3]	−1.5 [−2.7, −0.2]
**Genetic principal component 4**				
Median [IQR]	1.0 [−1.2, 3.3]	1.2 [−0.9, 3.5]	1.4 [−0.8, 3.8]	1.2 [−0.9, 3.6]

*Note:* Median and interquartile range (IQR) was reported for continuous variables, while absolute and relative frequencies were reported for categorical variables.

### 3.2 Association Between HLA‐E*01:01 and IM Diagnosis

We first assessed the association between HLA‐E*01 and the risk of IM diagnosis using a Cox model adjusted for demographic and genetic covariates, finding no statistically significant association (HR = 0.99 [95% CI: 0.94, 1.03], *p* = 0.56).

### 3.3 Association Between HLA‐E*01:01 and MS Diagnosis Stratified by IM Diagnosis

In the second analysis, we assessed the association between HLA‐E*01:01 and the risk of MS diagnosis, adjusting for the same covariates. Since the association between HLA‐DRB1*15:01 and MS risk violated the PH assumption (*p* < 0.05), we estimated separate HRs for ages ≤ 30 and > 30 (this threshold was chosen based on the Schoenfeld residuals plot shown in Figure S1, where HR decreases as age progresses and stabilizes around the age of 30). The Cox model revealed no statistically significant association between HLA‐E*01:01 and MS risk (HR = 1.06 [95% CI: 0.99, 1.13], *p* = 0.055).

We then included in the model the IM diagnosis as a time‐varying covariate along with its interaction terms with HLA‐E*01:01, HLA‐DRB1*15:01, and lack of HLA‐A*02:01 allele. The model was further adjusted for childhood body size and smoking. The results highlighted a statistically significant interaction between HLA‐E*01:01 allele and IM diagnosis (*β* = 0.50, [95% CI: 0.27; 0.66], *p*‐value < 0.001), implying that the associations between each factor with MS varied depending on the levels of the other. Specifically, in individuals without IM diagnosis, HLA‐E*01:01 was not significantly associated with MS (*p* = 0.051). Conversely, in individuals with IM diagnosis, MS risk significantly increased by 74% in heterozygotes (HLA‐E*01:01/01:03) and 301% in homozygotes (HLA‐E*01:01/01:01) (*p* < 0.001) (see Table 2 for HRs and 95% CIs). In line with previous findings [10, 11, 12], the Cox model also highlighted a statistically significant interaction between HLA‐DRB1*15:01 allele and IM diagnosis (but not between the absence of HLA‐A*02:01 allele and IM diagnosis). This highlights how HLA‐E*01:01 alleles further increase MS risk through their interaction with IM independently from the interaction already taking place between IM and HLA‐DRB1*15:01 alleles. Associations between IM diagnosis and MS given different combinations of HLA‐E*01:01 and HLA‐DRB1*15:01 alleles were fully described in Supporting Information and reported in Figure S2 and Table S3.

**TABLE 2:** Associations between HLA‐E*01 genotype and Multiple Sclerosis diagnosis in individuals without or with a history of Infectious Mononucleosis diagnosis based on a Cox model including the interaction between HLA‐E*01 and Infectious Mononucleosis.

	HR [95% CI]	*p*
**Without IM diagnosis**		
HLA‐E*01:03/01:03	Reference	
HLA‐E*01:01/01:03	1.05 [0.99, 1.08]	0.051
HLA‐E*01:01/01:01	1.11 [0.99, 1.17]	
**With IM diagnosis**		
HLA‐E*01:03/01:03	Reference	
HLA‐E*01:01/01:03	1.74 [1.36, 1.97]	< 0.001
HLA‐E*01:01/01:01	3.01 [1.81, 3.88]	

Abbreviations: 95% CI, 95% Confidence Interval obtained using bootstrap bias‐corrected accelerated method; HR, Hazard Ratio; IM, Infectious Mononucleosis.

### 3.4 Additive Interactions Between IM Diagnosis and HLA‐E*01:01

We also calculated interactions on the additive scale between HLA‐E*01:01 allele and IM diagnosis through the RERI and AP, with results reported in Table S4 and depicted in Figure S3. Results showed statistically significant additive interactions between HLA‐E*01:01 and IM diagnosis in increasing MS risk for any given combination of HLA‐DRB1*15:01 and HLA‐A*02:01 alleles (*p* < 0.001), with an estimated AP ≈ 40% in HLA‐E*01:01 heterozygotes and ≈ 65% in HLA‐E*01:01 homozygotes (see Supporting Information for further details).

### 3.5 Sensitivity Analyses

In the first sensitivity analysis, we censored the follow‐up up to 50 years of age to exclude 761 LOMS cases. In the second sensitivity analysis, we excluded 46,870 individuals born outside the UK. The results for both multiplicative and additive interactions between HLA‐E*01 and IM diagnosis were very similar to those in the main analysis. Multiplicative interactions were, respectively, *β* = 0.57 [95% CI: 0.28; 0.83] (*p* < 0.001), and *β* = 0.46 [95% CI: 0.24; 0.65] (*p* < 0.001), while additive interactions were reported in Table S5.

## 4 Discussion

Our study investigated the link between HLA‐E*01 polymorphisms, a history of IM diagnosis (a clinical manifestation of EBV infection) and MS risk, using data from 487,144 individuals in the UK Biobank. Previous research suggested that the HLA‐E*01:03 allele, compared to HLA‐E*01:01, enhances immune activation against EBV‐infected B cells, potentially protecting against MS [5]. However, GWASs have not identified a significant association between the HLA‐E*01 genotype and MS risk. Based on these insights, our study aimed to provide further investigation by using a statistical approach in a real‐world data setting. Specifically, we aimed to investigate whether HLA‐E*01:01 is linked to an increased risk of MS following the occurrence of an IM diagnosis, potentially due to the previously mentioned weaker immune control against EBV‐infected B cells [5].

Our findings indicate that HLA‐E*01:01 alone is not associated with an increased risk of MS or IM. Instead, when IM was diagnosed, individuals carrying the HLA‐E*01:01 allele had a significantly increased MS risk, with a proportion of combined risk attributable to interaction effects between HLA‐E*01:01 and IM estimated around 40% in heterozygotes and 65% in homozygotes [30]. The result supports our hypothesis, as it suggests a biologically relevant interaction between IM and HLA‐E*01:01, where the genetic variant predisposes to MS. Importantly, the interaction between IM and HLA‐E*01:01 in increasing MS risk acts independently from the interaction between IM diagnosis and HLA‐DRB1*15:01 (a well‐known MS risk allele), as the association between IM and MS was strongest in individuals carrying both HLA‐E*01:01 and HLA‐DRB1*15:01 alleles. In contrast, individuals who lacked both alleles showed no significant increase in MS risk following IM, implying that genetic predisposition may be necessary for IM to act as a risk factor for MS.

Overall, these findings suggest that HLA‐E*01 is associated with increased MS risk in EBV‐infected individuals diagnosed with IM, supporting our hypothesis and the findings by Vietzen et al. [5]. While previous studies focused on class II HLA alleles, such as HLA‐DRB1*15:01, this result highlights the need to incorporate the class I HLA‐E*01:01 allele when modeling multiple MHC components of MS risk, especially in the context of prior EBV infection. Specifically, future research should aim to identify HLA allele combinations that confer the highest MS susceptibility in the presence of EBV infection, ideally using detailed data on EBNA1 levels, to better capture EBV seroconversion and IM symptomatology. If confirmed, HLA‐E*01:01 screening, along with other MS‐related alleles, could improve MS risk assessment and become a key tool for targeted monitoring and early detection strategies in EBV‐exposed individuals, particularly those with a history of IM diagnosis, and who could benefit from closer monitoring for early intervention. Furthermore, the interaction between HLA‐E*01:01, IM, and MS risk suggests potential preventive and therapeutic strategies involving HLA‐E‐mediated immune responses to EBV.

While this study benefits from a large sample size, a long follow‐up that covers an individual's lifetime, extensive confounder adjustment, and a prospective design that reduces reverse causality concerns, several limitations must be acknowledged. The main limitation relates to the collection of IM cases through diagnoses based on healthcare records or self‐reporting a history of severe IM. Consequently, the prevalence of diagnosed IM cases in our cohort (0.8%) predominantly reflects cases requiring hospitalization or medical attention, which is lower than the estimated prevalence in the general population (≈10%) that also includes asymptomatic and mild cases [31]. This limitation implies that our findings are most applicable to severe, clinically recognized IM cases, and further research is needed to explore their generalizability to EBV seroconversion and milder IM symptomatology. MS diagnoses were also derived from healthcare records and self‐reports, some dating back decades, which may have introduced misclassification. Additionally, some early‐onset MS cases may have been missed due to mortality before study enrollment. However, the alignment of MS prevalence in the UK Biobank with national estimates suggests accurate case identification [32, 33]. Lastly, the study's predominantly white British population limits generalizability to other ethnic groups.

## Author Contributions

**Andrea Nova:** conceptualization, methodology, software, data curation, formal analysis, visualization, writing – original draft, writing – review and editing. **Davide Gentilini:** writing – review and editing. **Giovanni Di Caprio:** conceptualization, writing – review and editing. **Sonia Bourguiba‐Hachemi:** writing – review and editing. **Nicolas Vince:** writing – review and editing. **Pierre‐Antoine Gourraud:** writing – review and editing. **Luisa Bernardinelli:** writing – review and editing. **Teresa Fazia:** conceptualization, methodology, formal analysis, writing – review and editing, supervision.

## Conflicts of Interest

The authors declare no conflicts of interest.

## Supporting information


Data S1.

Data S2.

Data S3.

Figure S1.

Figure S2.

Figure S3.

Tables S1‐S5.

## Data Availability

Researchers may obtain access to the data used in this study upon reasonable request to the UK Biobank study team (https://www.ukbiobank.ac.uk/).
