# Supplementary material for: Stratifying Multiple Sclerosis Susceptibility Risk: The Role of HLA‐E*01 and Infectious Mononucleosis in a Population Cohort
Source: Eur J Neurol. 2025 Apr 7;32(4):e70131. doi: 10.1111/ene.70131 (PMC11973926; doi:10.1111/ene.70131)
Supplement: Supplementary file 2 — Data S2. [file ENE-32-e70131-s005.docx]

**Supplemental Material for “Stratifying MS Susceptibility risk: the role of HLA-E*01 and Infectious Mononucleosis in a Population Cohort”.**

**Methods**

**Outcome**

MS cases were defined according to the ICD-10 diagnosis code G35. Diagnosis date was retrieved as the earliest date from at least one source among hospital admissions, primary care, death registries and self-report (see Supplementary Table 1 for further details). To better characterize the date of MS onset and hence avoiding delayed MS diagnoses(21), we anticipated the date of MS diagnosis in case of an earlier diagnosis of: i) demyelination (ICD-10: G36 and G37), ii) abnormal findings on diagnostic imaging of central nervous system (ICD-10: R90), or iii) presence of other MS onset-related symptoms occurring in the 5 years prior to MS diagnosis date, including vision-related symptoms (such as optic neuritis) (ICD-10: H46, H47, H53, H55), sensory and motor symptoms (ICD-10: G25, G83, R20, R25, R26, R27, R29), bladder and bowel symptoms (ICD-10: K59, N31, N39), fatigue, dizziness, pain and weakness (ICD-10: G72, R42, R52, R53, M54, M62), dysphagia and speech problems (ICD-10: R13, R47, R49), depressive, sleep and memory disorders (ICD-10: F32, F33, G47, R41), epilepsy (ICD-10: G40), sexual dysfunction (ICD-10: F52), and disease of the spinal cord (ICD-10: G95). Age at MS diagnosis was determined by subtracting the date of birth from the date of MS diagnosis.

**Multiple Imputation**

Missing data for ethnicity (1.7%), place of birth (0.3%), having older siblings (1.5%), childhood body size at age 10 (2.1%), ever smoking on most or all days (0.5%), were imputed using multiple imputation by chained equations under the assumption that data were missing at random. Missing values were replaced specifying a predictive mean matching model along 15 iterations. The predictors used to impute missing data included genotype data, infectious mononucleosis (IM), confounders, and outcome data (a binary MS indicator and its Nelson-Aalen hazard function^1^).

**Statistical Interactions**

We investigated interactions on both additive and multiplicative scale among HLA-E*01 and IM diagnosis. On an additive scale, interaction means that the combined effects of two exposures is either greater or lesser than the sum of their individual effects, which, compared to the multiplicative scale, more closely corresponds to a mechanistic interaction^2^. Briefly, a mechanistic interaction implies that there are individuals for whom the outcome would occur if both exposures were present but not if only one or the exposures were present. This interaction suggests that individuals with combined exposures are characterized by an additional risk due to interaction effects which may be involved the biological mechanism underlying the disease. Moreover, evaluating interactions on the additive scale is considered more relevant in the context of disease prevention^3^, as it allows to identify the subgroup of individuals whom would benefit most from interventions. On the other hand, an interaction on a multiplicative scale implies that the combined effect of two exposures is either greater or lesser than the product of their individual effects. In simpler terms, this measure allows us to evaluate if the relative risks of one exposure vary within the levels of the other. However, contrary to the additive interaction, the multiplicative interaction alone does not inform us if the combination of two exposures produces an additional risk due to their interaction effects. Therefore, since both scales convey a different information, it is strongly recommended to present them both^4^.

To estimate additive and multiplicative interactions, we implemented a Cox including interaction terms between IM with HLA-E*01, HLA-DRB1*15 and HLA-A*02, adjusted for the other confounders:

$$\lambda\left( t| MS;X \right)= \lambda_{0} \left( t \right)*exp(\beta_{1}*IM+\beta_{2}*HLA-E:01+\beta_{3}*HLA-DRB1*15:01+\beta_{4}*HLA-A*02:01+\beta_{5}*IM*HLA-E:01+\beta_{6}*IM*HLA-DRB1*15:01 +\beta_{7}*IM*HLA-A*02:01+\beta_{8}*V)$$

Where $\lambda(t;MS;X)$ denotes the hazard rate given covariates $X$ (which includes IM, HLA alleles and other confounders), $\lambda_{0} \left( t \right)$ denotes the baseline hazard rate (in absence of IM diagnosis and other genetic/environmental factor $X$). $V$ denotes the baseline confounders and $\beta_{8}$ the vector for the respective coefficients. $\beta_{1}$ denote the principal effect for IM diagnosis, $\beta_{2}$ denote the principal effect for HLA-E*01:01 allele, $\beta_{3}$ denote the principal effect for HLA-DRB1*15:01 allele, and $\beta_{4}$ denote the principal effect for HLA-A*02:01 allele. $\beta_{5}$ denotes the multiplicative interaction term between diagnosis of IM and HLA-E*01 allele, $\beta_{6}$ denotes the multiplicative interaction term between diagnosis of IM and HLA-DRB1*15:01 allele, and $\beta_{7}$ denotes the multiplicative interaction term between diagnosis of IM and HLA-A*02:01 allele.

To evaluate the multiplicative interactions, we evaluated the parameters $\beta_{5}$, $\beta_{6}$ and $\beta_{7}$. $\beta_{5}>0$ implies a positive multiplicative interaction between HLA-E*01:01 and IM diagnosis, while $\beta_{5}<0$ a negative multiplicative interaction. A positive multiplicative interaction implies that the Hazard Ratio due to the presence of the first exposure is higher in the subgroup of individuals presenting the second exposure (and vice versa).

To estimate the associations between HLA-E*01 and MS based on the presence of IM diagnosis, we calculated the contrasts between coefficients as follows:

| **HLA-E*01** | **IM diagnosis** | **Hazard Ratio** |
| --- | --- | --- |
| 01/03 vs 03/03 | - | Exp($\beta_{2}$) |
| 01/01 vs 03/03 | - | Exp($\beta_{2}*2$) |
| 01/03 vs 03/03 | + | Exp($\beta_{2}+\beta_{5}$) |
| 01/01 vs 03/03 | + | Exp($\beta_{2}*2+\beta_{5}*2$) |

To estimate the associations between IM and MS based on the presence of HLA-E*01:01, HLA-DRB1*15:01, and HLA-A*02:01 alleles, we calculated the contrasts between coefficients as follows:

| **IM diagnosis** | **HLA-E*01:01** | **HLA-DRB1*15:01** | **HLA-A*02:01** | **Hazard Ratio** |
| --- | --- | --- | --- | --- |
| Yes vs No | - | - | + | Exp($\beta_{1}$) |
|  | - | + | + | Exp($\beta_{1}+\beta_{6}$) |
|  | + | - | + | Exp($\beta_{1}+\beta_{5}$) |
|  | + | + | + | Exp($\beta_{1}+\beta_{5}+\beta_{6}$) |
|  | - | ++ | + | Exp($\beta_{1}+\beta_{6}*2$) |
|  | ++ | - | + | Exp($\beta_{1}+\beta_{5}*2$) |
|  | ++ | ++ | + | Exp($\beta_{1}+\beta_{5}*2+\beta_{6}*2$) |
|  | - | - | - | Exp($\beta_{1}+\beta_{7}$) |
|  | - | + | - | Exp($\beta_{1}+\beta_{6}+\beta_{7}$) |
|  | + | - | - | Exp($\beta_{1}+\beta_{5}+\beta_{7}$) |
|  | + | + | - | Exp($\beta_{1}+\beta_{5}+\beta_{6}+\beta_{7}$) |
|  | - | ++ | - | Exp($\beta_{1}+\beta_{6}*2+\beta_{7}$) |
|  | ++ | - | - | Exp($\beta_{1}+\beta_{5}*2+\beta_{7}$) |
|  | ++ | ++ | - | Exp($\beta_{1}+\beta_{5}*2+\beta_{6}*2+\beta_{7}$) |

Additive interactions between IM diagnosis and HLA-E*01:01 given a specific combination of HLA-DRB1*15:01 and HLA-A*02:01 alleles were instead quantified calculating the Relative Excess Risk due to Interaction (RERI) using the formula:

$$\mathrm{RERI}\left( IM=Yes;HLA-E*01:01=x \right| HLA-DRB1*15:01=y; HLA-A*201=z)=\exp\left( \beta_{1}+\beta_{2}*x+\beta_{5}*x+\beta_{6}*y+\beta_{7}*z \right)-\exp\left( \beta_{1}+\beta_{6}*y+\beta_{7}*z \right)- \exp\left( \beta_{2}*x \right)+1$$

Where $x$ denotes the number of HLA-E*01:01 alleles (1 or 2), $y$ denotes the number of HLA-DRB1*15:01 alleles (1 or 2), $z$ denotes the presence or absence of HLA-A*02:01 allele (0 or 1). RERI can range from -∞ to ∞. In the absence of additive interaction, RERI=0. A $\mathrm{RERI}$>0 implies a positive additive interaction, while a $\mathrm{RERI}$<0 implies a negative additive interaction. Another measure of additive interaction used is the Attributable Proportion due to interaction (AP)^5^ and is defined as:

$$AP= \frac{\mathrm{RERI}\left( IM=Yes;HLA-E*01:01=x \right| HLA-DRB1*15:01=y; HLA-A*201=z)}{\exp\left( \beta_{1}+\beta_{2}*x+\beta_{5}*x+\beta_{6}*y+\beta_{7}*z \right)}$$

AP measures the proportion of MS risk in the group with $IM = “Yes”$ and $HLA-E*01:01=x$ that is due to the interaction itself. AP can range from -∞ to 1. In the absence of additive interaction, AP=0. AP> 0 implies super-additivity, while AP< 0 implies sub-additivity. An AP closer to 1 suggests that the additive interactions between the exposures is strong and therefore that individuals having both the exposures exhibits a higher risk compared to individuals not exposed or being exposed to one of the exposures only.

As mentioned above, additive interaction is deemed more appropriate to obtain insights on the presence of mechanistic interactions^6^. In this context, within Rothman’s sufficient cause framework^7^, a mechanistic interaction can be established between exposures $A$ and $B$ when $RERI>0$, assuming that: i) the confounding between exposures $A$ and $B$ with the outcome has been removed and that ii) neither $A$ or $B$ can ever pose as a protective factor for any individual (monotonicity assumption). Denoting with $D_{AB}$ the counterfactual outcome, i.e., the occurrence of the event, given that the individual had or had not been exposed to exposures $A$ and $B$, a mechanistic interaction, i.e., $RERI>0$, implies that there are some individual for whom$D_{A+B+}=1$and $D_{A+B-}=D_{A-B+}=0$^8^. Therefore, a mechanistic interaction (or synergism) allows to establish if certain individuals experience the outcome only if both exposures are present, but not if only one of the exposures is present. While these interactions can offer some clues about the underlying biology, these do not provide a direct way to translate the observed interactions from data into a clear understanding of the biological mechanisms involved, and therefore do not necessarily tell us anything about physical or functional interactions.

**References**

1. White IR, Royston P. Imputing missing covariate values for the Cox model. Stat. Med. 2009;28(15):1982–98.

2. Van Der Weele TJ, Knol MJ. A tutorial on interaction. Epidemiol. Method. 2014;3(1):33–72.

3. Greenland S. Interactions in epidemiology: Relevance, identification, and estimation. Epidemiology 2009;20(1):14–7.

4. Knol MJ, VanderWeele TJ. Recommendations for presenting analyses of effect modification and interaction. Int. J. Epidemiol. 2012;41(2)

5. Li R, Chambless L. Test for Additive Interaction in Proportional Hazards Models. Ann. Epidemiol. 2007;17(3):227–36.

6. Rothman KJ, Greenland S. Causation and causal inference in epidemiology. Am. J. Public Health 2005;95(Suppl 1):S144-50.

7. Rothman KJ, Greenland S, Walker AM. Concepts of interaction. Am. J. Epidemiol. 1980;112(4)

8. Vanderweele TJ, Robins JM. Empirical and counterfactual conditions for sufficient cause interactions. Biometrika 2008;95(1)
