# Supplementary material for: Stratifying Multiple Sclerosis Susceptibility Risk: The Role of HLA‐E*01 and Infectious Mononucleosis in a Population Cohort
Source: Eur J Neurol. 2025 Apr 7;32(4):e70131. doi: 10.1111/ene.70131 (PMC11973926; doi:10.1111/ene.70131)
Supplement: Supplementary file 3 — Data S3. [file ENE-32-e70131-s006.docx]

**Supplemental Material for “Stratifying MS Susceptibility risk: the role of HLA-E*01 and Infectious Mononucleosis in a Population Cohort”.**

**Results**

**Associations between IM diagnosis and MS diagnosis stratified by combinations of HLA alleles**

**Supplementary Figure 2** illustrates HRs with 95%CIs for IM-MS associations across HLA alleles combinations, stratified by age (up to or over 30 years), and full results, including p-values, were reported in **Supplementary Table 3**. While the association between IM and MS was not statistically significant in individuals lacking *HLA-E*01:01* and *HLA-DRB1*15:01* alleles (HR=0.85, [95%CI:0.68; 1.49], p-value=0.747), a statistically significant increase in MS risk was estimated in individuals carrying two copies of the *HLA-DRB1*15:01* alleles, both up to 30 years of age (HR=4.42, [95%CI:1.43; 6.49], p-value=0.023) and over 30 years of age (HR=1.58, [95%CI:1.44; 2.76], p-value=0.001), and in individuals further carrying two copies of the HLA-E*01:01 allele, both up to 30 years of age (HR=11.34, [95%CI:3.50; 16.42], p-value<0.001) and over 30 years of age (HR=4.06, [95%CI:3.10; 7.18], p-value<0.001). These results highlight how *HLA-E*01:01* alleles further increase MS risk through their interaction with IM, independently of the interaction already taking place between IM and *HLA-DRB1*15:01* alleles (and vice versa).

**Additive interactions between IM diagnosis and HLA-E*01:01**

We calculated interactions on the additive scale between *HLA-E*01:01* allele and IM diagnosis through the RERI and AP. **Supplementary** **Figure 3** depicts the RERIs with 95% CIs across HLA alleles combinations, stratified by age (up to or over 30 years), and full results, including, p-values, HRs, and APs, were reported in **Supplementary Table 4**. Results showed statistically significant additive interactions between *HLA-E*01:01* and IM diagnosis for any given combination of *HLA-DRB1*15:01* and *HLA-A*02:01* alleles (p<0.001). As for multiplicative interactions, additive interactions were very similar regardless of the presence or absence of the *HLA-A*02:01* allele. Conversely, these increased in magnitude given a higher number of *HLA-DRB1*15:01* alleles. Specifically, the lowest RERI was observed in IM individuals carrying one copy of *HLA-E*01:01* allele, but lacking *HLA-DRB1*15:01* and *HLA-A*02:01* alleles (RERI=0.54 [95%CI:0.43, 0.75], p-value<0.001). In contrast, the highest RERI was found in IM individuals up to 30 years of age carrying two copies of both *HLA-E*01:01* and *HLA-DRB1*15:01* alleles, as well as the *HLA-A*02:01* allele (RERI=8.79 [95%CI:2.83, 13.94], p-value<0.001). Lastly, the AP was ≈40% in *HLA-E*01:01* heterozygotes and ≈65% in *HLA-E*01:01* homozygotes given any *HLA-DRB1*15* and *HLA-A*02* combination.

**Figure Legends**

**Supplementary Figure 1:** Schoenfeld residuals plot used to check the proportional hazard assumption between HLA-DRB1*15:01 and Multiple Sclerosis risk.

**Supplementary Figure 2:** Associations between Infectious Mononucleosis and Multiple Sclerosis risk in individuals with different combinations of HLA alleles (E*01:01, DRB1*15:01, and A*02:01) and with an age up to or over 30 years old, based on a Cox model including interaction terms between HLA alleles and Infectious Mononucleosis. For E*01:01, DRB1*15:01, and A*02:01, – denotes the absence of the allele. For A*02:01, + denotes the presence of at least one allele, while for E*01:01 and DRB1*15:01 it denotes the presence of one allele only. For E*01:01 and DRB1*15:01, ++ denotes the presence of two alleles. The labels indicate the Hazard Ratios with 95% confidence intervals between brackets.

**Supplementary Figure 3:** Additive interactions measured using the Relative Excess Risk due to Interaction (RERI) between HLA-E*01 and Infectious Mononucleosis on Multiple Sclerosis risk in individuals with different combinations of HLA alleles (E*01:01, DRB1*15:01, and A*02:01) and with an age up to or over 30 years old, based on a Cox model including interaction terms between HLA alleles and Infectious Mononucleosis. For E*01:01, DRB1*15:01, and A*02:01, – denotes the absence of the allele. For A*02:01, + denotes the presence of at least one allele, while for E*01:01 and DRB1*15:01 it denotes the presence of one allele only. For E*01:01 and DRB1*15:01, ++ denotes the presence of two alleles. The labels indicate the RERIs with 95% confidence intervals between brackets.
