# Supplementary figures and images for: Stratifying Multiple Sclerosis Susceptibility Risk: The Role of HLA‐E*01 and Infectious Mononucleosis in a Population Cohort
Source: Eur J Neurol. 2025 Apr 7;32(4):e70131. doi: 10.1111/ene.70131 (PMC11973926; doi:10.1111/ene.70131)

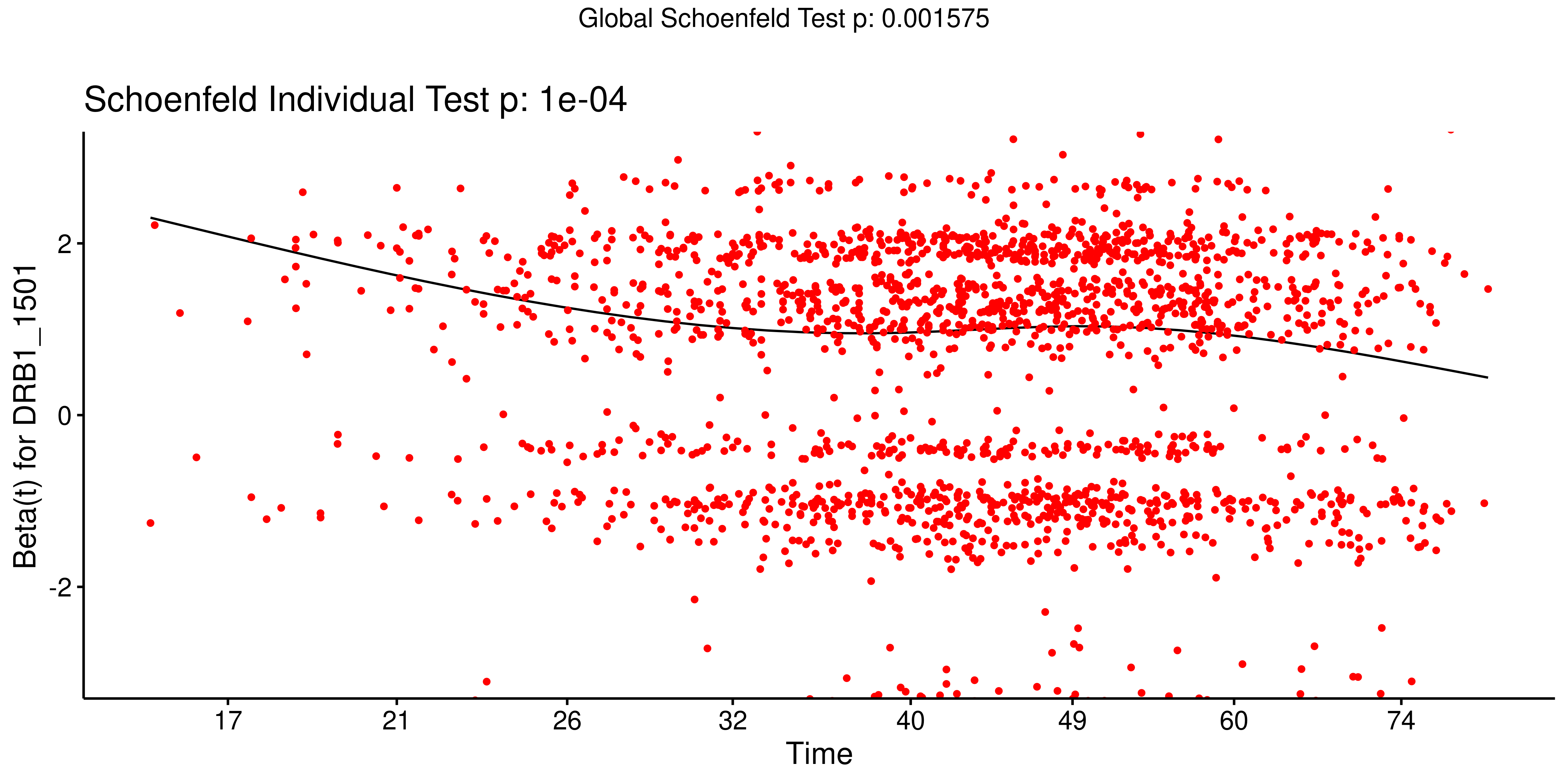

Supplement: Supplementary file 4 — Figure S1. [file ENE-32-e70131-s001.tif]

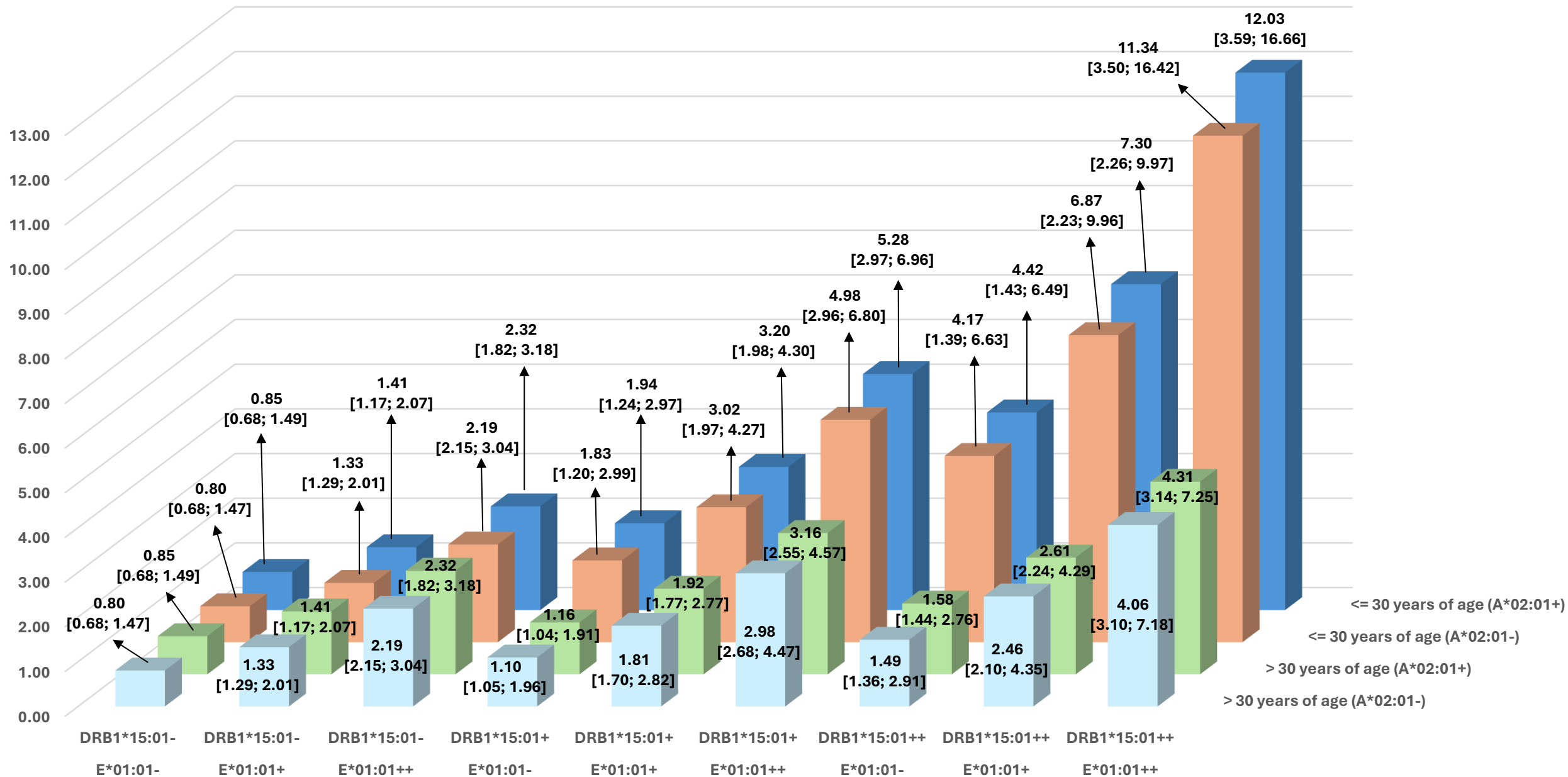

Supplement: Supplementary file 5 — Figure S2. [file ENE-32-e70131-s004.pdf]

RERI

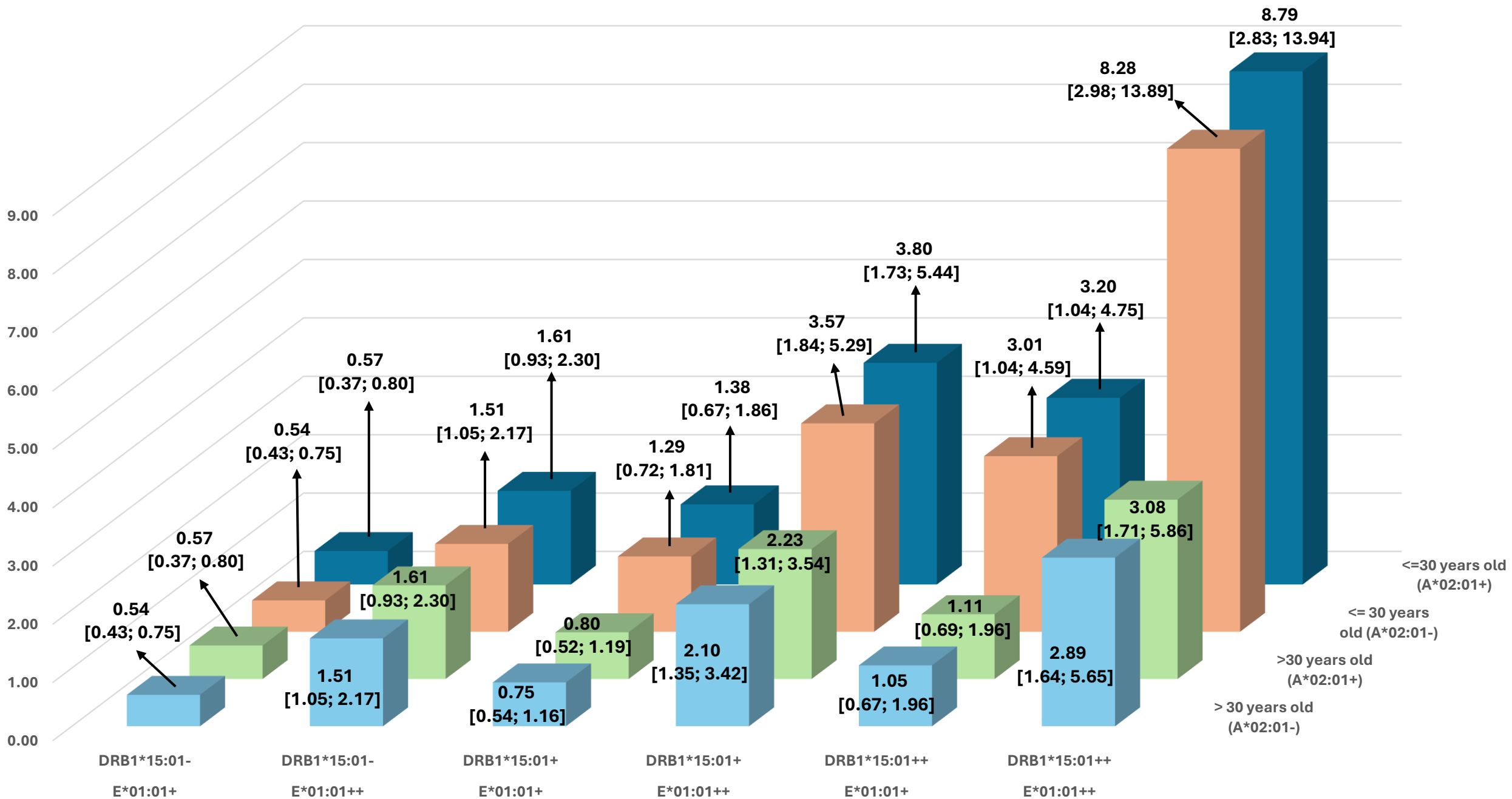

Supplement: Supplementary file 6 — Figure S3. [file ENE-32-e70131-s002.pdf]
